# The Solution to the Waste Problem

**Published:** 1912-01-13

**Authors:** 


### INSTITUTIONAL HOUSEKEEPING.

(Contributions invited: Workers' Questions will be welcome.)

### The Solution of the Waste Problem.

THE art of cheeseparing has been an unpopular one from time immemorial. In England especially a certain large-handedness is a tradition of the housekeeper, and to be "near," which ought to be a term of praise, has degenerated into the reproach attaching to one who has the disagreeable trick of providing just too little for the company. In its proper sense, however, the housekeeper must be always aiming at this quality of "nearness." She must not calculate to the last ounce the amount of food she puts on the table, so that if appetites are extra sharp someone goes short. But she must be near in the sense of shaving her cheeses so that good food is not thrown to the pigs on the wasteful rind, of peeling her potatoes lightly, of using every fragment of good food snipped off or left over in the kitchen and liable to be counted against the household economy.

### THE HOUSEKEEPER'S QUALITIES.

The two qualities needed by the housekeeper in this connection are watchfulness and forethought. It is watchfulness which has its eyes everywhere and sets a spirit of economy stirring among everyone whose duties lie in the distribution of the food. It is forethought which plans matters in advance so that not only are fragments saved but they are also turned to the best possible account. It is small use to save every scrap of bread and every pound of dripping if in the end no better use can be found for them than the "drip and swill" bucket, sold for a few pence to whoever will be at the pains to call for it, or to some contractor who already makes good money out of the institution.

The process of saving these by-products of the commissariat undoubtedly demands the attention of a skilled housekeeper at liberty to devote her entire time to her own department, and it is the duty of all managers of institutions to recognise that unless they provide a trained housekeeper (not a probationer in training) to control the victualling of the establishment, waste and inefficiency must ensue. Rarely indeed can the matron, on whom rest all the weighty responsibilities of patients, nursing staff, and administration, secure sufficient time in the course of her daily routine to train the kitchen staff, to test samples of provisions and check the supplies, to prepare well-thought-out menus for the different sections of the household, and to keep so close a watch on the distribution of necessaries that all are conscious that waste of any article will be instantly detected. We have had the privilege of knowing more than one matron who did succeed in achieving splendid success in domestic matters as well as in general administration without the aid of a housekeeper, but in every case it was at the expense of so much nervous energy that the age for retirement was forestalled by many years, and the hospital managers, in their premature loss of invaluable services, were guilty of the cruel blunder of killing the goose who laid the golden egg.

We believe that the discussion of methods for avoiding waste must demonstrate beyond all question the necessity for the trained housekeeper in every institution containing as many as 100 beds. Let no one think that the details of how to utilise such insignificant articles as crusts of bread, of dripping and bones, are beneath the dignity of a scientific paper. In domestic matters nothing is common or unclean. The aim of good housekeeping is to carry efficiency and economy into every nook and cranny, and the battle which is waged over the crust may prove of good service in training the combatants for further victories.

Expense is the usual plea for dispensing with a housekeeper and allowing the over-burdened matron to cope as best she may with the problems of the commissariat. It may be well to examine the outcome of this laudable thirst for economy. An establishment containing 100 beds for patients will be made up of not fewer than 150 persons all told, including patients, nursing staff, domestics, etc. Let it be conceded that each member of such a household will be responsible for the waste of even so little as ½ lb. of bread, 2 oz. of fat, and ¼ lb. of bones, etc., in the course of a week. The amount really wasted of these commodities per head in the kitchen alone is demonstrably greater than this in even very economically managed institutions. But we have taken it at the lowest estimate. The amount would work out as follows:—

| | £ | s. | d. |
|---|---|---|---|
| 150 half-pounds of bread at ½d. | 0 | 6 | 3 |
| 150 two ounces of fat at ½d. | 0 | 6 | 3 |
| 150 quarter-pounds of bones, etc., at ¼d. | 0 | 3 | 1 |
| Total for the week | 0 | 15 | 7 |
| Total for the year | £40 | 10 | 4 |

### THE "INSTITUTIONAL ATTITUDE."

The real value of the bones, trimmings, and fat, if properly utilised in the institution, is considerably more than that estimated. But be that as it may, it will be seen that a clever housekeeper in a comparatively small hospital could easily pay her own salary out of what she saved in only a few directions. When it is realised that these are among the smallest economies which could be effected, it will be seen without further argument that the institution which is bent on feeding its inmates generously at the minimum cost cannot afford to dispense with a trained housekeeper. The first step to reform, however, is to get rid of the traditional institution attitude. This may be summed up as a conviction that the waste in hospitals is enormous, but that it always takes place in *the other hospitals.*

[We regret owing to pressure on our space that many questions and answers have been unavoidably held over.]

